# Evaluating the Efficacy of Levetiracetam on Non-Cognitive Symptoms and Pathology in a Tau Mouse Model

**DOI:** 10.3390/biomedicines12122891

**Published:** 2024-12-19

**Authors:** Jackson C. Thompson, Marselina Levis Rabi, Michelle Novoa, Kevin R. Nash, Aurelie Joly-Amado

**Affiliations:** Department of Molecular Pharmacology and Physiology, Morsani College of Medicine, University of South Florida, 12901 Bruce B Downs Blvd, Tampa, FL 33612, USA; jacksonthompson723@gmail.com (J.C.T.); mlevisrabi@usf.edu (M.L.R.); michellentbusiness@gmail.com (M.N.); nash@usf.edu (K.R.N.)

**Keywords:** tau, mouse models, levetiracetam, Alzheimer’s disease

## Abstract

**Background/Objectives:** Alzheimer’s disease (AD) is marked by amyloid-β plaques and hyperphosphorylated tau neurofibrillary tangles (NFTs), leading to cognitive decline and debilitating non-cognitive symptoms. This study aimed to evaluate compounds from four different classes in a short-term (7-day) study using transgenic tau mice to assess their ability to reduce non-cognitive symptoms. The best candidate was then evaluated for longer exposure to assess non-cognitive symptoms, cognition, and pathology. **Methods:** Tg4510 mice, expressing mutated human tau (P301L), were administered with levetiracetam, methylphenidate, diazepam, and quetiapine for 7 days at 6 months old, when pathology and cognitive deficits are established. Drugs were given in the diet, and non-cognitive symptoms were evaluated using metabolic cages. Levetiracetam was chosen for longer exposure (3 months) in 3-month-old Tg4510 mice and non-transgenic controls to assess behavior and pathology. **Results:** After 3 months of diet, levetiracetam mildly reduced tau pathology in the hippocampus but did not improve cognition in Tg4510 mice. Interestingly, it influenced appetite, body weight, anxiety-like behavior, and contextual fear memory in non-transgenic animals but not in Tg4510 mice. **Conclusions:** While levetiracetam has shown benefits in amyloid deposition models, it had limited effects on tau pathology and behavior in an animal model of tau deposition, which is crucial for AD context. The differential effects on non-transgenic versus Tg4510 mice warrant further investigation.

## 1. Introduction

Alzheimer’s disease (AD) is the most prevalent form of dementia and is characterized by worsening cognitive symptoms over time. AD is primarily age related, as 96% of the 6.9 million cases recorded in 2023 occurred in adults aged 65 and older [1]. Other predispositions for developing AD include genetics, sex, and environmental factors [2,3,4]. The downstream effects of these factors result in a buildup of extracellular amyloid-β (Aβ) plaques and intracellular neurofibrillary tangles (NFTs) in the brain, comprising the phosphorylated tau (pTau) protein [5]. Protein aggregation and neuroinflammation ultimately leads to neurodegeneration and cognitive impairments [6].

AD is accompanied not only by cognitive decline but also by non-cognitive symptomatology, commonly referred to as behavioral and psychological signs and symptoms of dementia (BPSD) [7]. BSPD changes in AD lead to a poorer quality of life and can be life-threatening [8,9]. BSPDs also remain important predictors of caregiver burden and depression, which is another facet of the disease that cannot be overlooked. Despite their profound consequences, most non-cognitive changes in AD remain poorly studied. Weight loss is a non-cognitive symptom that is commonly reported in AD patients [10] and is now recognized as a clinical feature of AD [11,12,13,14,15,16], correlated with accelerated progression of the disease, a higher rate of institutionalization and increased mortality [8,17,18,19]. Increased physical activity levels such as wandering or excessive pacing are also reported as BPSD [20]. Lower body weight and increased feeding behavior have also been described in mouse models of amyloid or tau deposition [21,22,23,24,25] together with increased energy expenditure [24] or hyperactivity [25].

Therefore, in this study, we first tested different classes of pharmacotropic drugs for their ability to reduce non-cognitive symptoms in Tg4510 mice [26]. Levetiracetam (anticonvulsant), methylphenidate (psychomotor stimulant), diazepam (anxiolytic), and quetiapine (antipsychotic) were tested on non-cognitive symptomology in 6-month-old Tg4510 mice over a period of 7 days. The drug that was most efficient at reducing non-cognitive symptoms was then chosen for long-term (3 months) preventative treatment in 3-month-old mice.

Tg4510 mice are a cross between two established mouse lines that results in a mouse that produces a mutated human tau (P301L) in the forebrain that can be suppressed with doxycycline. This mutation can be found in heredity dementia [27], and is associated with NFT formation in humans as well as in these mice. Tg4510 mice exhibit progressive accumulation of tau pathology and cognitive impairments. In addition, we previously described that these mice develop progressive hyperactivity compared to non-transgenic littermates [28]. This hyperactive phenotype was correlated with a failure to increase body weight which was associated with decreased food intake efficiency and a loss of white adipose tissue. Resting metabolism was also significantly increased in aging Tg4510 mice [28]. 

The outcome of our short-term study using 4 different pharmacotropic drugs indicated that levetiracetam was best suited for the long-term study. This result was consistent with the literature in AD patients as well as previous studies in animal models of amyloid deposition, showing potential benefits of anticonvulsant drugs. Indeed, AD patients are more likely to develop seizures than healthy controls [29,30,31]. Similarly, evoked seizures in animal models of AD can precipitate cognitive decline and worsen AD pathology [32]. There is evidence that one of the underlying mechanisms of these seizures is excessive neuronal excitability and neurotoxicity observed in the AD brain [33]. These observations led to the investigation of antiepileptic drugs as a putative therapeutic avenue to prevent neuronal hyperexcitability and possibly slow down the progression of AD symptoms [34]. Levetiracetam (Lev) has been used as an antiepileptic drug since its approval by the food and drug administration (FDA) in the year 2000. Lev is used alone or in conjunction with other medications for the treatment of diverse types of seizures. Compared to phenytoin, the most commonly prescribed AED (anti-epileptic drug), Lev is more effective at treating convulsive status epilepticus (CSE) and is just as effective at treating children with acute repetitive seizures (ARS) [35]. In addition, Lev has less severe adverse side effects than other AEDs, however, it has been associated with some adverse neurological and psychiatric effects [36,37,38]. Lev, through studies and off label usage, has been used to treat a wide range of neurological conditions, including multiple sclerosis, Tourette’s syndrome, and autism spectrum disorder [39,40,41]. The exact mechanism of levetiracetam is still under investigation, but it is well understood that synaptic vesicle protein 2A (SV2A) is a levetiracetam binding site [42,43]. SV2A is an integral membrane protein responsible for trafficking neurotransmitters and facilitating their release into the synapse via Ca^2+^ mediated exocytosis [44,45]. Levetiracetam can also act as a voltage-gated Calcium channel blocker when added to rat hippocampal slices [46]. This mechanism was shown to inhibit excitatory signaling in the hippocampus of epileptic rats which explains levetiracetam’s antiepileptic activity [47]. SV2A is colocalized with synaptophysin and synaptotagmin, where these proteins facilitate vesicle exocytosis into the synapse [48]. It is theorized that levetiracetam reaches SV2A by either permeating the cell membrane or binding to an SV2A exposed to the extracellular space during vesicle membrane fusion [49,50]. The exact effect of levetiracetam on SV2A is unknown, but it is postulated that levetiracetam modulates SV2A to a more favorable conformation [51]. Other roles of SV2A are still under investigation, as levetiracetam improves mitochondrial function, which is diminished upon the knockout of SV2A [52]. In addition, levetiracetam seems to play a role in modulating GABAergic responses to increase inhibitory signals in the brain [53]. Levetiracetam has also been found to interact with AMPA receptors, which are glutamate-gated ion channels found on post-synaptic ganglion [54] and to increase PSD-95, a post-synaptic scaffolding protein found mainly in glutamate neurons, which is an indicator of increasing synaptic plasticity [55,56]. 

Levetiracetam has recently been explored as a potential treatment for AD, due to its safety profile and potential positive effects on various neurological disorders. Sanchez et al. found that in hAPP mice, an AD mouse model that overproduces Aβ and exhibits epileptiform activity [57,58], treatment with Lev reversed hippocampus (HPC) remodeling, behavior abnormalities, synaptic dysfunction, and learning deficits [59]. Das et al. found similar findings of restored neural network function in hAPP mice treated with Lev, in addition to the suppression of inflammation-related gene expression [60]. This group also gave Lev (300 mg/kg body weight/day) to treat pathogenic tau-producing mice (hTau-A152T) for 6 months and found treatment decreased irregular brain rhythms [61], an effect which was also seen to a lesser extent in non-transgenic (Ntg) controls. Alavi et al. performed daily injections of Lev (100 and 150 mg/kg) in rats with streptozotocin-induced dementia, which reduced pTau expression in the HPC [62]. One month of administration also improved cognitive symptoms and biochemical markers of inflammation and oxidative stress. Finally, Zhang et al. treated APP23/MAPT crossbred mice with Lev and kainic acid (KA—induces Aβ and tau hyperphosphorylation) for 3 months. Lev was found to improve learning and memory mechanistically through the dephosphorylation of tau via GSKα/β and CDK5 pathways [63]. Despite its promising impact on amyloid pathology, the influence of levetiracetam on tau-related pathways and non-cognitive manifestations remain unclear, particularly in tauopathy-specific models. Hence, the presented study aimed at studying the effects of levetiracetam exposure in Tg4510 mice, a mouse model of tau deposition.

The results of short-term exposure to levetiracetam, methylphenidate, diazepam, and quetiapine in old Tg4510 mice are presented and demonstrate that levetiracetam was the best candidate to be assessed in a longer period paradigm.

## 2. Materials and Methods

### 2.1. Animals

Testing procedures in animals received approval from the Institutional Animal Care and Use Committee at the University of South Florida and were conducted in compliance with the eighth edition of the ‘Guide for the Care and Use of Laboratory Animals’, published by the National Academy of Science, National Academies Press, Washington, DC (2011) [64]. Tg4510 mice were bred as previously outlined [26,65] and following IACUC breeding protocols. The mice were housed together (up to 5 animals in the same cage in accordance with IACUC guidelines) in a twelve-hour light/dark cycle, with food and water provided ad libitum. The breeding of the mice was staggered to facilitate behavior analysis in cohorts, balanced for gender, genotype, and treatment. Drugs were purchased from Millipore Sigma (St. Louis, MO, USA) with the following references: methylphenidate (1433008), diazepam (D0899), quetiapine (Y0001657), levetiracetam (L8668). 

### 2.2. Experimental Design

#### 2.2.1. Short Term Study

Tg4510 mice aged 6 months and age-matched non-transgenic littermates were randomly assigned to treatment or placebo groups with sample sizes of 6. Drugs were administered in food for delivery (based upon pilot studies and the literature), namely 40 mg/kg/d for LEV [66]; 10 mg/kg/d for MPH [67]; 0.5 mg/kg/d for diazepam [68]; and 5 mg/kg/d for quetiapine [69,70]. On day 0, mice were single housed in metabolic caging (Phenomaster, TSE systems). The Phenomaster is a comprehensive multi-caging system platform that allows the non-invasive recording of food intake, drink intake, daily activity, and the evaluation of metabolism. More specifically, each cage was equipped with a gas analyzer that recorded oxygen consumption (VO_2_) and carbon dioxide production (VCO2) to determine the animal’s energy expenditure and respiratory exchange ratio (RER) by indirect calorimetry. On day 4, mice were given drug diets while in the Phenomaster cages for 7 days. Monitoring stopped 4 days after the end of the drug treatment for a total of 15 days. On day 15, mice were returned to their housing facility. The tissue was harvested the following day.

#### 2.2.2. Long-Term Study

Tg4510 mice and non-transgenic littermate mice were equally randomly assigned to be treated (40 mg/kg/day levetiracetam) or placebo (control chow diet) groups (*n* = 10, 5F and 5M). All mice were single housed, and food intake and body weight were recorded once a week for 3 months. During the last 2 weeks before tissue collection, the following behavior tests were performed: open field, Y-maze, RAWM with reversal, Rotarod, and fear conditioning. Following the completion of behavioral testing, the mice were euthanized, and the tissue was collected.

### 2.3. Behavioral Testing

Behavior testing was completed in cohorts of mice balanced for gender, genotype, and treatment by an experimenter blind to animal treatment and genotype. Prior to each behavior test, mice were single housed in temporary cages for acclimation and for the duration of the test, being only identifiable by their ear punches.

To assess general locomotor activity, anxiety, and exploratory behavior, an open field test was used. Animals were placed in a 40 cm square arena for 15 min during which movement was video tracked using ANY-maze software Version 7.8 (ANY-maze, Stoelting, Wood Dale, IL, USA). Total distance of travel was measured as general activity levels, and anxiety-like behavior was assessed by time spent in the center/perimeter. 

Motor coordination, balance, and endurance were evaluated using a rotarod apparatus (Stoelting, Wood Dale, IL, USA). Mice were placed on a rotating rod, starting at an initial rotation of 4RPM with a gradual acceleration to 40 RPM over 5 min. Mice were required to match the speed of the rotating rod to avoid falling. The duration each mouse stayed on the rod (latency to fall) was recorded across four trials per day over two consecutive days.

Radial arm water maze (RAWM) was performed to assess learning and memory, as published previously [71]. Briefly, this maze consisted of 6 swim arms extending from the central start area with a hidden escape platform located at the end of one of the arms. The test arm was different for each mouse but remained constant in each trial. Mice were trained for 15 trials on day 1, where the platform alternated between visible and hidden. On day 2, all 15 trials were performed with a hidden platform and the starting arm was different for each trial. Incorrect arm entries counts were considered as errors. Failure to make an arm entry within 15 s was also scored as an error. On the third day, a reversal trial was performed to assess the mouse’s ability to establish a new strategy. The goal platform was placed in the arm 180° from the original location. Mice were given 15 trials, all with a hidden platform. The errors for the blocks of 3 consecutive trials were averaged for data analysis.

Associative learning and memory were assessed using conditioned fear response test. During the training phase, mice were placed in a conditioning chamber (Stoelting, Wood Dale, IL, USA) for 7 min, where they experience an auditory stimulus (a 30 s noise at 70 dB) paired with an aversive stimulus (a 0.5 mA mild foot shock) during the last 2 s of the tone. This pairing occurred at 3 min and 6 min to establish the association. To assess contextual fear conditioning, mice were returned to the same chamber without the aversive stimulus 24 h later. For cued fear conditioning, the context of the chamber was carefully changed (floor, walls, and olfactory cues) and mice were exposed to auditory stimulus without the foot shock. In all conditions, movement was recorded through video tracking (ANY-maze software, Stoelting, Wood Dale, IL, USA). Fear response was evaluated by measuring freezing behavior.

### 2.4. Tissue Collection

Tissue collection was performed as described in [72]. Mice aged 6 months were injected with a solution containing pentobarbital and phenytoin, then transcardially perfused with PBS. The right hemisphere was dissected and immediately frozen for biochemistry analysis while the left hemisphere was immersed for 24 h in 4% phosphate-buffered paraformaldehyde for fixation. The fixed hemispheres were cryoprotected in gradient sucrose solutions (up to 30%) for 24 h each. The brains were then frozen on a cold stage of a sliding microtome and sectioned horizontally into 25 μm slices. Brain slices were stored in Dulbecco’s phosphate-buffered saline with 10 mM sodium azide at 4 °C.

### 2.5. Histopathology

Gallyas’ silver stain was performed as described [73]. Tissue sections were then digitally scanned using a microscope Axio Scan Z.1 (Zeiss Inc., Oberkochen, Germany). The quantification of positively stained areas in the regions of interest was performed using NearCYTE software (http://nearcyte.org) [74,75]. Values from 8 sections per mouse were averaged to yield a single value for that region, which was then used in subsequent statistical analysis.

### 2.6. Biochemical Analyses

Tissues for Western blot analysis were prepared as previously described [76]. Equal amounts of proteins according to BCA (5 μg/well for soluble fraction, 1 μg/well for insoluble fraction) were loaded in each well of a 4–12% Tris glycine gel and transferred to a 0.2 μm pore size nitrocellulose membrane and immunoblotted with H150 (Anaspec, Fremont, CA, USA), SV2A (Santacruz biotechnology, Dallas, TX, USA), pSer396-tau (Anaspec, Fremont, CA, USA), actin (Sigma Aldrich, St Louis, MO, USA), pSer199/202 (Anaspec, Fremont, CA, USA), synaptotagmin (Proteintech, Rosemond, IL, USA), PSD95 (Millipore-Sigma, Burlington, MA, USA), and synaptophysin (Abcam, Waltham, MA, USA), at a 1:1000-fold dilution. Secondary antibodies tagged with fluorescent dye (IRDye 800CW, IRDye 680CW, LI-COR Biosciences, Lincoln, NE, USA) were used at a dilution of 1:10,000. Western blot quantification was performed using densitometric analysis with the Odyssey imaging system (LI-COR Biosciences, Lincoln, NE, USA) as detailed previously [72]. Markers from different molecular weights or different hosts of primary antibodies (allowing the use of secondary antibodies tagged with different fluorescent probes) were probed on the same gel, seepSer199/202 and synaptotagmin probes in Section 3. 

### 2.7. Quantification of Levetiracetam in Biological Samples

Levetiracetam concentration was determined in plasma and brain samples using HPLC analysis, performed at the CPAS platform at USF, based on [77] and using a Kinetex C18 Polar 4.6 × 100 mm and 2.6 μm column. Briefly, 40 µL of methanol were added to plasma aliquots (100 µL) or tissue homogenate supernatant (150 µL) for protein precipitation. Then, 10 µL of the internal standard (IS) working solution (50 µg/mL) in acetonitrile and 1 mL of ethyl acetate were also added, vortex-mixed for 30 s, and centrifuged at 12,045× *g* (3 min for plasma and 5 min for tissues samples) in order to extract the drug and the IS. The resulting upper organic layer was transferred to a clean glass tube and the aqueous layer was re-extracted using liquid–liquid extraction procedure. To concentrate the analytes, both organic phases were combined and evaporated under a gentle nitrogen stream at 45 °C. The resulting solid residue was reconstituted in 100 µL of water and acetonitrile mixture (90:10, *v*/*v*) by vortex-mixing for approximately 1 min and sonication in an ultrasonic bath at room temperature for approximately 1 min. Afterwards, the extracts were transferred to a 0.22 µm Costar^®^ Spin-X centrifugal filter (Corning, Inc., Corning, NY, USA), centrifuged at 12,045× *g* for 3 min or 5 min (plasma and tissues, respectively). Lastly, 20 µL of the final filtered samples were injected into the HPLC system. Validation parameters are shown in Table 1.

### 2.8. Statistical Analysis and Rigor

The data were examined using ANOVA or repeated measures ANOVA, followed by Fisher’s protected least significant difference post hoc tests (Graphpad software Version 10.1.0, San Diego, CA, USA). A *p*-value of less than 0.05 was deemed significant in all analyses. Unless stated otherwise, all data are shown as mean values ± standard error of the mean (SEM).

The investigators pledge that they conducted this project with the utmost integrity, accountability, and transparency. To ensure rigor for this experimental design, mice were assigned to treatment conditions and given a study number using a random number generator, researchers were blinded to the animal groups during testing, with animals being identified only by a unique identifier number (ear punches). Animals from different genotypes but with the same treatment were housed together (up to 5 animals per cage).

Treatments were rendered blind to the experimenter by color coding the cage cards allowing rigorous monitoring of food intake and body weight.

Additional measures were taken during behavior testing to avoid the identification of treatment group (see Section 2.3).

## 3. Results

### 3.1. Reduction in Non-Cognitive Symptoms by Pharmacotropic Drugs in a Mouse Model of Tauopathy

To determine which class of compound was better suited to decrease non-cognitive symptomology, we first evaluated the effects of four different FDA approved pharmacotropic drugs in 6-month-old Tg4510 mice, when pathology, cognitive, and non-cognitive symptoms are well established. We chose compounds from four different drug classes based on the literature. Levetiracetam (Lev, Keppra) is an anticonvulsant drug known to decrease seizures and kindling in several animal models of epilepsy [78]. It is approved for the treatment of partial-onset epileptic seizures. Levetiracetam is further known to block CNS seizure activity in mice with amyloid deposition [59] and improve cognition in MCI cases with elevated hippocampal activity [79]. Levetiracetam reduces neurotransmitter release via the inhibition of voltage-operated K^+^ currents, N-type Ca^2+^ channels and binding to synaptic vesicle protein 2A [80]. Methylphenidate (MPH, Ritalin) is a psychomotor stimulant beneficial in the treatment of attention deficit hyperactivity disorder (ADHD) [81]. MPH is a competitive substrate for noradrenaline and dopamine transporters and inhibits their reuptake. In rodents, MPH increases exploration and locomotion in wild type mice but can reduce hyperactivity in ADHD models [82]. A first clinical trial on AD patients with MPH (ADMET) showed improvement in attention and apathy [83]; however, neither cognition nor hyperactivity was evaluated. A second phase of this clinical study ADMET revealed that changes in apathy symptoms were not consistently correlated with changes in global cognition, selective attention, or working memory, as relationships differed in MPH and placebo groups. These results are consistent with the view that apathy as a syndrome is related to, but distinct from, cognition [84]. Diazepam (Valium) is an antianxiety/CNS depressant drug of the benzodiazepine family. Our laboratory found a decrease in Aβ deposition in a mouse model of AD (Tg2576) upon treatment with diazepam [68]. Diazepam possesses sedative, hypnotic, anxiolytic, anticonvulsant, and muscle relaxant properties. Diazepam is a positive allosteric modulator of the alpha subunit of GABA-A receptor. Quetiapine (Seroquel) is an antipsychotic approved for the treatment of schizophrenia and bipolar disorder and has sedative effects [85]. In an amyloid mouse model (APP/PS1), memory impairment and oxidative stress were attenuated with quetiapine [86]. Quetiapine further decreased glial activation [87] and pro-inflammatory cytokines in APP/PS1 mice [69]. Quetiapine also induced weight gain in comparative studies [87]. Quetiapine is a dopamine, serotonin, histamine, and adrenergic antagonist [88].

Levetiracetam (Lev), methylphenidate (MPH), diazepam (Dia), and quetiapine (Quet) were administered through food as a delivery system (based upon pilot studies and the literature), namely 40 mg/kg/d for Lev [89]; 10 mg/kg/d for MPH [67]; 0.5 mg/kg/d for Dia [68]; and 5 mg/kg/d for Quet [86] and were tested on non-cognitive symptomology in 6-month-old Tg4510 mice over a period of 7 days, using metabolic cages (Phenomaster, TSE systems, Missouri, MO) that allow the non-invasive recording of food intake, drink intake, daily activity, and the evaluation of metabolism by indirect calorimetry.

**Figure 1:**
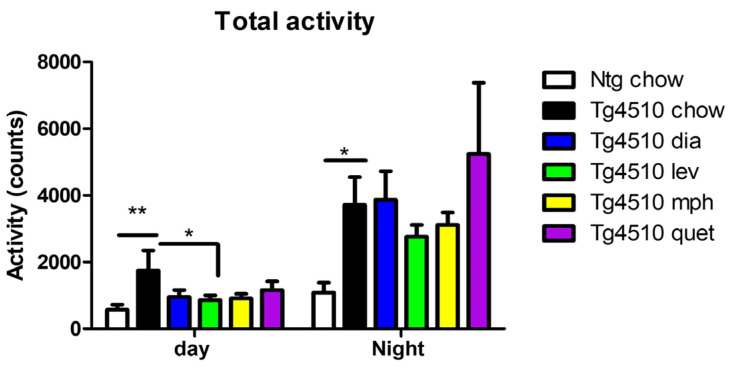
Total activity (counts) during the last 3 days of drug treatment at night and during the day in 6-month-old non-transgenic mice fed a control diet (ntg chow, white bar), and age-matched Tg4510 mice fed a control diet (chow, black bars) or a diet with diazepam, 0.5 mg/kg/d (dia, blue bars); levetiracetam, 40 mg/kg/d (lev, green bars); methylphenidate, 10 mg/kg/d (mph, yellow bars); or quetiapine, 5 mg/kg/d (quet, purple bars). Data are presented as mean ± SEM; *n* = 5–8/group. Statistical comparisons were performed using a two-way ANOVA followed by Fisher’s post hoc test: * *p* < 0.05, ** *p* < 0.01.

**Figure 2:**
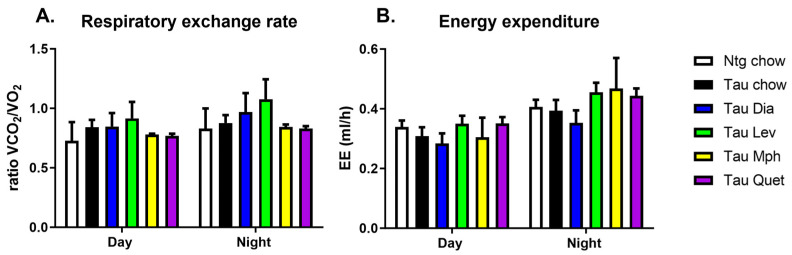
Respiratory exchange rate, i.e., ratio of volume of CO_2_ produced of O_2_ consumed, (**A**) and energy expenditure (**B**) at night and during the day in 6-month-old non-transgenic mice fed a control diet (Ntg chow, white bar), and age-matched Tg4510 mice fed a control diet (chow, black bars) or a diet with diazepam, 0.5 mg/kg/d (Dia, blue bars); levetiracetam, 40 mg/kg/d (Lev, green bars); methylphenidate, 10 mg/kg/d (Mph, yellow bars); or quetiapine, 5 mg/kg/d (Quet, purple bars). Data are presented as mean ± SEM, *n* = 5–8/group. Statistical comparisons using two-way ANOVA followed by Fisher’s post hoc test.

**Figure 3:**
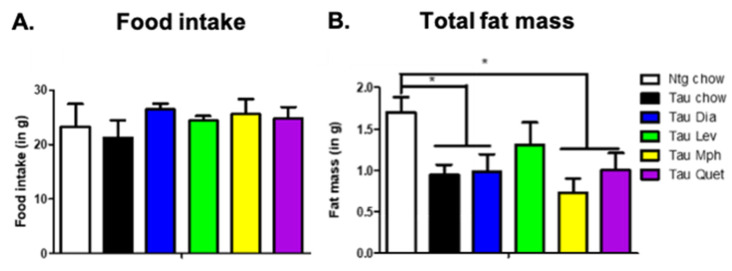
Weekly food intake (**A**) over the course of drug treatment and total fat mass (**B**) at the end of the treatment in 6-month-old mice non-transgenic mice fed a control diet (ntg chow, white bar) and age-matched Tg4510 mice fed a control diet (chow, black bars) or a diet with diazepam, 0.5 mg/kg/d (Dia, blue bars); levetiracetam, 40 mg/kg/d (Lev, green bars); methylphenidate, 10 mg/kg/d (Mph, yellow bars); and quetiapine, 5 mg/kg/d (Quet, purple bars). Data are presented as mean ± SEM, *n* = 5–8/group. Statistical comparisons using two-way ANOVA followed by Fisher’s post hoc test: * *p* < 0.05.

As we showed previously [28], Tg4510 mice given the control diet displayed significant increased activity during the day and at night when compared to non-transgenic littermates (Figure 1). Treatment with diazepam, methylphenidate, or quetiapine, although inducing trends towards decreases in activity during the day, did not induce significant changes in activity when compared to tau mice fed a control diet. However, Tg4510 mice treated with levetiracetam showed significantly decreased activity compared to Tg4510 mice fed a control diet, especially during the day (Figure 1). Respiratory exchange rate or respiratory quotient is the ratio of CO_2_ produced to O_2_ consumed and is representative of the energetic fuel being metabolically used. No changes were observed between Tg4510 mice fed a control diet when compared to non-transgenic mice fed a control diet either during the day or at night (Figure 2A). Treatment with diazepam, methylphenidate, levetiracetam, or quetiapine did not induce significant changes in respiratory exchange rate (Figure 2A). No changes were observed in energy expenditure between Tg4510 mice fed a control diet when compared to non-transgenic mice fed a control diet either during the day or at night (Figure 2B). Treatment with diazepam, methylphenidate, levetiracetam, or quetiapine did not induce significant changes in energy expenditure either (Figure 2B). There were no differences in food intake over the course of treatment between the distinct groups (Figure 3A), indicating that the lack of changes in the parameters observed could not be due to an aversion to the diet from the mice.

As previously described, we observed a significant decrease in total fat mass in Tg4510 mice fed a chow diet when compared to non-transgenic control mice (Figure 3B). This significant difference was maintained in Tg4510 mice treated with diazepam, methylphenidate, or quetiapine, but not levetiracetam. Indeed, a trend towards an increase in total fat mass was observed in Tg4510 mice treated with levetiracetam even though it did not reach significance in comparison with Tg4510 control mice fed a chow diet. This trend in the normalization of body mass could be explained by the significant reduction in the hyperactive phenotype observed in Tg4510 mice fed a Lev diet. We did not observe a difference in body mass, brown adipose tissue, or total muscle mass among groups (Table 2). A significant difference was observed in brain weight between Tg4510 and non-transgenic mice, regardless of treatment (Table 2).

In conclusion of this short-term study, among the four drugs evaluated, levetiracetam, an anti-convulsant, was the only drug able to significantly reduce the hyperactivity phenotype observed in 6-month-old Tg4510 mice both at night and during the day, following a short time exposure. There was also a trend towards the normalization of total fat mass in Tg4510 mice treated with levetiracetam that could be explained by the observed reduction in activity. Therefore, levetiracetam was considered for a longer period of treatment in a preventative paradigm.

### 3.2. Decreased Body Weight and Food Intake in Non-Transgenic but Not Tg4510 Mice, Following 3 Months of Diet with Levetiracetam

Three-month-old Tg4510 mice and their non-transgenic littermates were given either control diet or levetiracetam diet 40 mg/kg/day for 3 months. At the age of 5.5 months, the mice underwent a series of behavioral tests to assess cognition and non-cognitive symptoms (see Section 3.3). At 6 months, tissue was collected.

**Figure 4:**
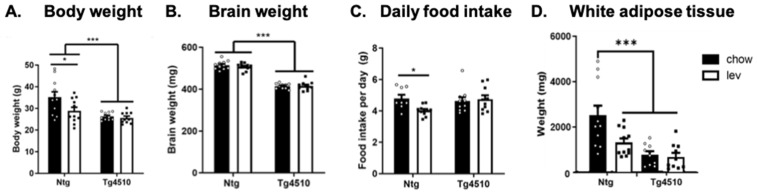
(**A**) Body weight, (**B**) brain weight, (**C**) food intake, and (**D**) adipose tissue weight comparisons between Ntg and Tg4510 mice fed chow (black bars) and a levetiracetam diet (lev, white bars). Statistical comparisons using two-way ANOVA followed by Fisher’s post hoc test: * *p* < 0.05, *** *p* < 0.001. Data are represented as means ± SEM.

Following 3 months of treatment, Tg4510 mice had a significantly lower body weight and brain weight than non-transgenic mice (Figure 4A,B), regardless of treatment. However, we found that non-transgenic mice fed levetiracetam weighed significantly less than non-transgenic mice fed a control diet (Figure 4A). There was no difference in food intake between non-transgenic mice and Tg4510 mice fed a control diet (Figure 4C). Interestingly, levetiracetam treatment significantly reduced food intake in non-transgenic mice but not in Tg4510 mice when compared to mice fed a regular diet (Figure 4C). This could indicate a new role for levetiracetam in the regulation of food intake and, more importantly, a discrepancy in the levetiracetam pathway in the brain of Tg4510 mice compared to non-transgenic mice. In addition, the decreased body weight in non-transgenic mice fed the levetiracetam diet was associated with a decrease in adipose tissue when compared to non-transgenic mice fed a regular diet (Figure 4D). Tg4510 mice fed a regular diet exhibited less adipose tissue when compared to non-transgenic controls, as previously shown by us; however, the levetiracetam diet did not affect this phenotype. There were no differences in muscle mass.

### 3.3. Behavior Analysis

**Figure 5:**
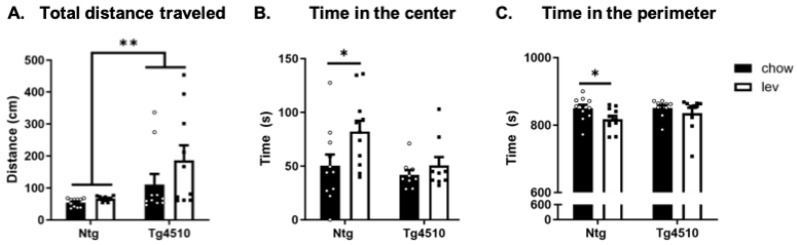
Open field test: (**A**) total distance traveled, (**B**) time in center, and (**C**) time in perimeter for Ntg and Tg4510 mice fed chow (black bars) and Lev diet (white bars). Statistical comparisons using two-way ANOVA followed by Fisher’s post hoc test: * *p* < 0.05, ** *p* < 0.01. Data are represented as means ± SEM.

Open field testing was used to assess locomotor activity and anxiety-like behavior in mice [90]. As expected, Tg4510 mice traveled significantly further than non-transgenic mice in open field testing; however, levetiracetam treatment had no effect (Figure 5A). No significant genotypic differences were found in the time spent at the perimeter or the time spent in the center. However, non-transgenic mice fed levetiracetam spent significantly less time at the perimeter and more time in the center than non-transgenic mice fed a control diet (Figure 5B,C), which is indicative of an anxiolytic effect.

**Figure 6:**
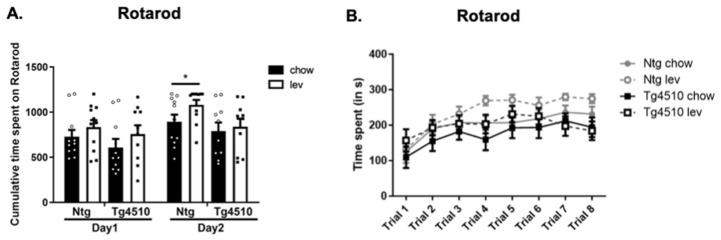
Latency to fall during Rotarod test represented as total time (**A**) and as trials (**B**) in Ntg and Tg4510 mice fed chow (black bars) and Lev diet (white bars). Statistical comparisons using two-way ANOVA followed by Fisher’s post hoc test and repeated measures ANCOVA: * *p* < 0.05. Data are represented as means ± SEM.

Next, motor coordination was assessed using the rotarod test [91]. Latency to fall from the rod was used to evaluate the rodents’ balance, coordination, and motor skills. We observed no significant differences between genotypes on day 1, regardless of treatment. During day 2, we observed a significant increase in cumulative time spent on the rotarod in non-transgenic mice treated with levetiracetam when compared to non-transgenic, control diet-fed animals (Figure 6A,B). This could be due to the fact that the mice undergoing Lev treatment weighed less than the controls (Figure 4A) or due to an effect of Lev on endurance. However, levetiracetam had no effect on Tg4510 mice.

**Figure 7:**
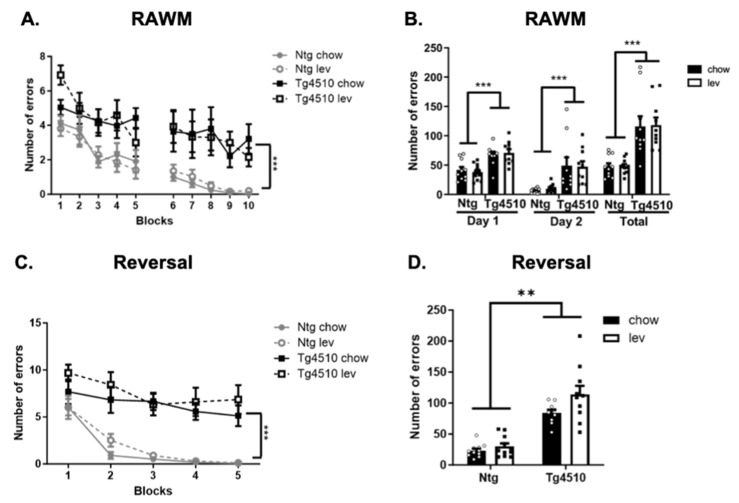
Radial arm water maze represented as blocks, with the average of number of errors per three trials (**A**) and total number of errors (**B**), and reversal represented as blocks (**C**) and total errors (**D**) in non-transgenic and Tg4510 mice fed chow (black bars) and a Lev diet (white bars). Statistical comparisons using two-way ANOVA followed by Fisher’s post hoc test or repeated measures ANOVA: ** *p* < 0.01, *** *p* < 0.001. Data are represented as means ± SEM.

Next, learning and memory were assessed using radial arm water maze and reversal [71]. During both RAWM (Figure 7A,B) and reversal (Figure 7C,D), Tg4510 mice made significantly more errors in the process of reaching the platform than non-transgenic mice, regardless of treatment. Levetiracetam had no effect during these tests.

**Figure 8:**
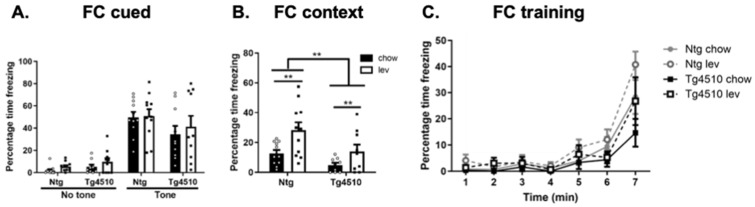
Cued (**A**) and contextual (**B**) fear conditioning tests with training (**C**) in Ntg and Tg4510 mice fed chow (black bars) and Lev diet (white bars). Statistical comparisons using two-way ANOVA followed by Fisher’s post hoc test or a repeated measures ANOVA: ** *p* < 0.01. Data are represented as means ± SEM.

Cued and contextual fear conditioning tests were then used to determine hippocampal vs. amygdala recall memory [92]. As previously shown, both genotypes recalled amygdala associated fear memory, as indicated by the increased time freezing when hearing the cued tone, regardless of treatment (Figure 8A). During contextual fear conditioning, Tg4510 mice fed a control diet spent significantly less time freezing than non-transgenic controls, which is indicative of impaired memory (Figure 8B). Levetiracetam diet significantly increased freezing time in both non-transgenic and Tg4510 mice (Figure 8B). All groups performed similarly during training (Figure 8C).

### 3.4. Levetiracetam Content

**Figure 9:**
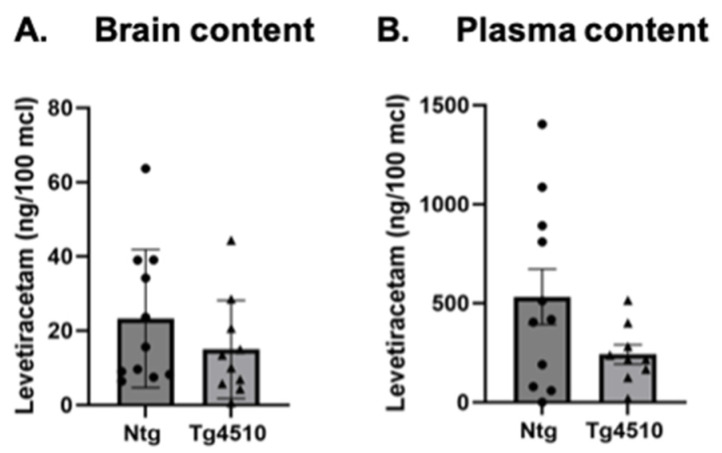
Levetiracetam content in the brain (**A**) and plasma (**B**) following 3 months of treatment in Ntg and Tg4510 mice fed a Lev diet. Statistical comparisons using Student’s *t*-test. Data are represented as means ± SEM.

We verified the brain and plasma content of levetiracetam-treated animals. We found no significant difference in the levetiracetam content either in the brain or in the plasma between non-transgenic and Tg4510 mice (Figure 9A,B).

### 3.5. Treatment with Levetiracetam Induced a Mild Decrease in Phosphorylated Tau

**Figure 10:**
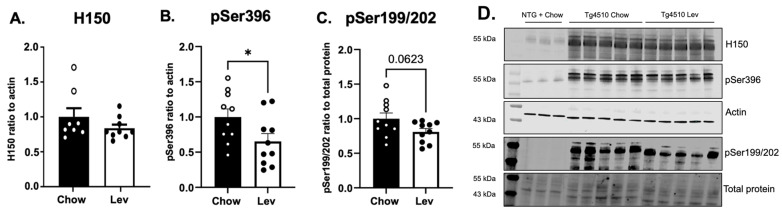
(**A**) Quantification of band densitometry of total tau (Tau 46, H150), (**B**) tau phosphorylated at serine 396, and (**C**) serine 199 and 202 in the hippocampus of Tg4510 mice fed chow (black) and mice fed a Lev diet (white). (**D**) Micrograph representation of Western blotting with H150, pSer396, and pSer199/202 (pTau) markers. Statistical comparisons using *t*-test. Data are represented as means ± SEM. * *p* < 0.05.

**Figure 11:**
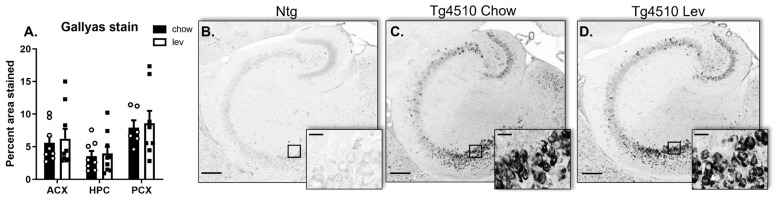
Quantification (**A**) of positive area stained with Gallyas’ silver stain in the anterior cortex (ACX), hippocampus (HPC), and posterior cortex (PCX) in Tg4510 mice fed a control chow diet (black bars) or a levetiracetam diet (white bars) for 3 months. Micrographic representation of Gallyas’ silver stain (Nissl) in non-transgenic (Ntg) (**B**) and Tg4510 mice fed with a control chow diet (**C**) or a levetiracetam diet (**D**) for 3 months. Statistical comparisons using two-way ANOVA followed by Fisher’s post hoc test. Data are represented as means ± SEM. Scale bar 100 μm for main picture and 20 μm for insert.

Then, we assessed tau pathology by measuring tau levels. The Western blot analysis of the hippocampus revealed a decreasing trend in total Tau and phosphorylated Tau at Ser199/202 in Tg4510 mice treated with levetiracetam when compared to controls. Levetiracetam treatment induced a statistically significant but mild reduction in the levels of pTau ser396 (Figure 10A). In addition, this reduction did not translate into a decrease in neurofibrillary tangles since the levels of Gallyas’ staining were equivalent in both controls and levetiracetam-treated animals (Figure 11).

### 3.6. Levetiracetam Normalized Levels of SV2A in Tau Mice

**Figure 12:**
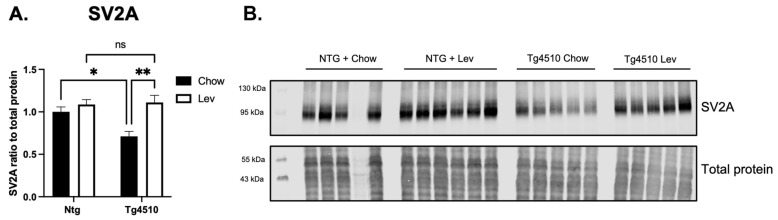
(**A**) Quantification of band densitometry for synaptic vesicle glycoprotein 2A (SV2A) in the hippocampus of Tg4510 mice fed chow (black) and mice fed a Lev diet (white). (**B**) Micrograph representation of Western blotting for SV2A and control proteins. Data are represented as means ± SEM. Statistical comparisons using two-way ANOVA followed by Tukey’s post hoc test. * *p* < 0.05, ** *p* < 0.01, ns: not significant.

**Figure 13:**
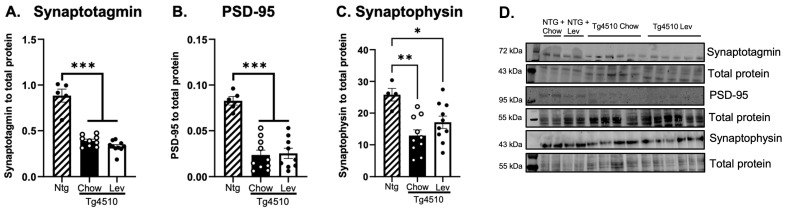
Quantification of band densitometry for synaptotagmin (**A**), PSD-95 (**B**), and synaptophysin (**C**) in the hippocampus of Tg4510 mice fed chow (black) and mice fed a Lev diet (white). Micrograph representation of Western blotting and total protein (**D**). Statistical comparisons using one-way ANOVA followed by Tukey’s post hoc test * *p* < 0.05, ** *p* < 0.01, *** *p* < 0.001. Please note, synaptotagmin was probed on the same gel as pSer199/202 (Figure 10); thus, the same total protein was used. We found no differences in non-transgenic mice fed a control diet or treated with Lev; therefore, these groups were pooled together for analysis.

To assess changes in the levetiracetam pathway, we measured levels of SV2A, synaptophysin, synaptotagmin, and PSD-95 in the hippocampus. There was a significant decrease in SV2A protein in Tg4510 mice when compared to non-transgenic mice, and this deficit was reduced with levetiracetam (Figure 12A,B). Tg4510 mice had a significant decrease in synaptic proteins synaptotagmin (Figure 13A,D), PSD-95 (Figure 13B,D), and synaptophysin (Figure 13C,D) when compared to non-transgenic mice, regardless of treatment.

## 4. Discussion

In our short-term experiment, although quetiapine has shown to have benefits in amyloid animal models in terms of reduced inflammation and improvement in memory at the dose used in this study [69,70], we did not identify significant changes in locomotor activity following short exposure in tau mice. However, in previous studies, animals were treated for 8 months with quetiapine, so we cannot exclude the possible effect of quetiapine on tau pathology and phenotype over a longer period of treatment. Methylphenidate was shown to improve non-cognitive symptoms in 5xFAD mice at the dose used in this study [67]. Although we observed a trend towards the restoration of the hyperactive phenotype when methylphenidate was given to tau mice for 7 days, it did not reach significance. This difference in outcome could be due to the different models used in this study and also the mode of administration. Indeed, in the study by Schneider et al., the drug was given as a bolus injection 45 min prior to testing, which could result in a higher peak of dopamine in response to MPH than the peak observed in mice fed a control diet. We previously showed that diazepam reduced amyloid pathology in the Tg2576 model at the dose used in this study [68]. Others have shown effects of diazepam on anxiety behavior and hyperactivity in a different animal model [93]. However, under the conditions of our experiment, we did not identify any differences in diazepam-treated mice in terms of basal activity. The role of levetiracetam in basal activity and body weight is poorly understood. The results of our short-term study show a reduction in the hyperactive phenotype in Tg4510 mice, which induced an increasing trend in the fat mass of these mice. Thus, we expected more pronounced changes in the activity, body weight, and fat mass of Tg4510 mice as a result of long-term treatment with levetiracetam. Interestingly we observed no changes in these parameters during the long-term study at the same dose. Moreover, levetiracetam treatment induced body weight loss in non-transgenic animals. This could indicate a differential effect of Lev between short-term treatment and longer-term treatment and should be considered for future studies in patients.

Our long-term study shows that levetiracetam had differential effects on different genotypes. Indeed, in non-transgenic mice, levetiracetam treatment led to a decrease in food intake and body weight or, more particularly, a decrease in white adipose tissue, together with increased endurance, as indicated by the increased time spent on the rotarod. However, these effects were not observed in Tg4510 mice. These results are novel and call for the further investigation of the role of levetiracetam in models where body weight and fat content are increased, such as obesity and diabetes. Similarly, we observed an anxiolytic effect of levetiracetam in non-transgenic animals, as described in patients in other studies [94], but not in Tg4510 mice. These results indicate that the levetiracetam pathway is disrupted in tau mice. This could be because Tg4510 mice exhibited lower levels of SV2A in the hippocampus, a known actor of the levetiracetam pathway [52,80]. These results are consistent with studies conducted in patients with AD were decreased levels of SV2A have been observed [95]. Interestingly, SV2A levels were normalized upon treatment with levetiracetam in Tg4510 mice, but this was not sufficient to restore the levetiracetam biological responses observed in non-transgenic mice. Other studies have demonstrated the increased expression of SV2A upon levetiracetam treatment [96,97]. This suggests a potential levetiracetam resistant state in tau mice, the mechanism of which is still unclear and requires further investigation. In addition, we did not observe a restoration of the levels of synaptotagmin, synaptophysin, or PSD95, which are required for SV2A action on synaptic plasticity, explaining why we did not observe cognitive improvements in this study [48,55,56]. Similarly, the mild decrease observed in phosphorylated tau in the hippocampus of Tg4510 mice treated with levetiracetam was not sufficient to improve cognition.

It is important to note the lack of consensus on the levetiracetam dose between different animal models. The 40 mg/kg dose chosen for this experiment was based on previous pharmacokinetics/pharmacodynamics studies performed in a mouse animal of AD (5XFAD) which suggested the use of a dose between 30 and 56 mg/kg for animal models for AD with an aggressive pathology [66]. Zheng et al. found that injecting a low dose of levetiracetam (50 mg/kg/day) was the most effective at reversing cognitive deficits, while a high dose (200 mg/kg/day) was the best at reducing Aβ aggregation [63]. However, Alavi et al. found that a high dosage (150 mg/kg/day injection) of levetiracetam was the most effective at ameliorating memory and learning deficits in streptozotocin-induced dementia but was ineffective at a low dose (50 mg/kg/day) [62]. In addition, this high dosage of levetiracetam decreased levels of phosphorylated (pSer396) tau when the low dose had no significant effect. Sanchez et al. tested the acute ip injection of several doses of levetiracetam (5, 50, 200 mg) and subcutaneous administration (75 mg/kg, 150 mg/kg) in hAPP mice and found that levetiracetam 75 mg/kg/day (IP injection) was effective at improving cognitive function and decreasing Aβ in hAPP mice [59]. It is important to note, however, that varying mouse models of AD differ in underlying pathology and therefore are subject to variability in response to levetiracetam treatment. Therefore, more dose–response studies are needed, particularly in tauopathy models, in order to decipher the effects of levetiracetam.

Recent clinical trials have investigated the potential of levetiracetam in treating Alzheimer’s disease (AD) [98,99]. A randomized, double-blind, placebo-controlled study found that levetiracetam was well-tolerated and improved spatial memory and executive function in AD patients with epileptiform activity, although it did not improve the primary outcome measure [98]. Another pilot study demonstrated levetiracetam’s safety and tolerability in AD patients without seizure history, but found no significant cognitive improvements [100]. A prospective observational study of levetiracetam monotherapy in advanced AD patients with new-onset seizures reported that 72% of participants remained seizure-free for at least one year [101]. While these findings suggest the potential benefits of levetiracetam in specific AD subgroups, particularly those with epileptiform activity or seizures, further research is needed to fully assess its efficacy in improving cognition and determine optimal treatment approaches for AD patients [98,99].

Here, we demonstrated that levetiracetam had a limited effect on tau pathology and no effect on cognition in a tau depositing model. We suggest that this lack of effect could be due to a levetiracetam-resistant state in tau mice. Indeed, the normalization of SV2A levels after levetiracetam treatment was not sufficient to restore the drug’s biological effect on synaptic plasticity. These results should be considered in ongoing or future clinical trials on AD where both amyloid and tau pathology are present. Recent research indicates that tau protein is more closely correlated with cognitive decline than amyloid plaques in Alzheimer’s disease, since amyloid plaques can accumulate in the brain for decades before symptoms appear. In contrast, the spread of tau neurofibrillary tangles (NFTs) is strongly associated with neuronal loss and cognitive deficits [102]. Studies have shown that elevated levels of phosphorylated tau in the brain correlate with the severity of cognitive decline and the progression of Alzheimer’s disease [103,104]. This suggests that tau pathology, rather than amyloid accumulation, plays a more direct role in the mechanisms leading to cognitive deterioration. Although further studies are required in tau models, especially to establish a dose–response relationship, our study, which shows that levetiracetam failed at decreasing neurofibrillary tangles and improving cognition in a tau model, could explain why the levetiracetam treatment of AD patients did not reach efficacy in cognitive outcomes.

In addition, our results show an effect of levetiracetam on body weight and food intake in the non-transgenic animals but not in our tau deposition mouse model. Levetiracetam’s effect on body weight remains controversial. Indeed, a large-scale analysis of clinical trials found no significant weight changes in patients treated with levetiracetam compared to treatment with placebo [105]. However, subsequent studies have reported conflicting results. Two children experienced significant weight loss whilst under treatment with levetiracetam, necessitating its cessation [106]. Similarly, 19 cases of dramatic weight loss were reported in adults taking levetiracetam at doses of 500–2000 mg/day [107]. A retrospective observational study of 1.1 million adult patients found that levetiracetam was associated with significant weight gain (mean 1.00 kg, *p* = 0.02) [108]. These conflicting findings suggest that levetiracetam’s effects on body weight may vary among individuals, and further research is needed to understand the factors contributing to these differences.

## 5. Conclusions

In conclusion, our study demonstrates that levetiracetam exhibits differential effects based on genotype, particularly in food intake and body weight. Additionally, an anxiolytic effect was observed in non-transgenic mice. However, these effects were absent in Tg4510 mice, suggesting a disruption in the levetiracetam pathway in tau mice. This disruption may be attributed to lower SV2A levels in the hippocampus of Tg4510 mice, a key component of the levetiracetam pathway. Although SV2A levels were normalized with levetiracetam treatment in Tg4510 mice, this did not restore the biological responses seen in non-transgenic mice, suggesting a levetiracetam-resistant state in these mice, which calls for further investigation. 

Future research should focus on elucidating the mechanisms underlying the disrupted levetiracetam pathway in tau mice. Investigating alternative strategies to enhance SV2A function or compensatory pathways in Tg4510 mice could provide insights into improving the efficacy of levetiracetam. More particularly, future studies could focus on combined therapies, such as dietary supplementation, which has been shown to potentiate levetiracetam’s effect on neuroprotection [109], or the use of brivaracetam, a levetiracetam analog with higher affinity to SV2A [110]. Additionally, exploring the impact of the levetiracetam/SV2A pathway on other tauopathy models and its long-term effects on cognitive function and neuroprotection will be crucial. Understanding the interaction between levetiracetam and other molecular targets in the brain may also reveal potential combinatory treatments to mitigate tau-related pathologies.

## Figures and Tables

**Table 1:** Main validation parameters of the HPCS-DAD method applied to quantify levetiracetam in plasma and brain matrices. LLOQ, lower limit of quantification; CV, coefficient of variation; Bias, deviation from nominal value. ^a^ Values expressed in µg/g. ^b^ Inter-day values, *n* = 5. ^c^ Equation of the calibration curve is given by the general equation of y = mx + b, with m corresponding to the slope and b to the intercept. The equation represents the peak areas signals of levetiracetam to that of the internal standard (y) versus the corresponding plasma concentration of levetiracetam (x). ^d^ Weighted linear regression using 1/x^2^ as the best weighting factor.

Validation Parameter	Plasma	Brain
Calibration range (μg/mL)	2.5–40	2.5–160 ^a^
Regression equation ^b,c^	y = 0.022641x – 0.013985	y = 0.037154x – 0.013824
Coefficient of determination (r^2^) ^d^	0.9948	0.9920
LLOQ (μg/mL)	2.5	0.625
Inter-day		
Precision (%CV)	2.88–9.48	5.79–9.42
Accuracy (%Bias)	−1.52–13.42	−4.7–2.07
Intra-day		
Precision (%CV)	2.81–9.91	2.17–6.33
Accuracy (%Bias)	0.60–8.88	−7.61–4.45
Recovery (%)	58.86–79.18	69.10–87.07

**Table 2:** Body weight (BW), brain weight, brown adipose tissue weight (BAT), and total muscle mass (as the sum of soleus, gastrocnemius, and extensor digitorum longus) of 6-month-old mice given treatment for 7 days and switched to chow diet for 5 days before euthanasia. Non-transgenic mice fed a control diet (Ntg chow) and age-matched Tg4510 mice fed a control diet (Tg4510 chow) or diet with diazepam, 0.5 mg/kg/d (Tg4510 Dia); levetiracetam, 40 mg/kg/d (Tg4510 Lev); methylphenidate, 10 mg/kg/d (Tg4510 Mph); and quetiapine, 5 mg/kg/d (Tg4510 Quet). Data are presented as mean ± SEM, *n* = 5–8/group. ^a^ *p* < 0.001 against non-transgenic group.

	Ntg Chow	Tg4510 Chow	Tg4510 Dia	Tg4510 Lev	Tg4510 Mph	Tg4510 Quet
BW (g)	30.39 ± 0.58	27.475 ± 0.92	27.66 ± 1.01	28.17 ± 0.78	27.46 ± 0.62	29.60 ± 0.67
Brain weight (mg)	476.60 ± 2.23	392.17 ± 1.0 ^a^	392.33 ± 1.21 ^a^	392.83 ± 1.35 ^a^	393.40 ± 1.86 ^a^	392.20 ± 0.43 ^a^
BAT (mg)	149.80 ± 2.95	126.17 ± 2.94	138.33 ± 4.54	167.17 ± 4.99	143.80 ± 3.61	132.80 ± 2.75
Total muscle (mg)	282.40 ± 1.60	270.50 ± 1.76	275.67 ± 2.54	280.67 ± 1.68	255.60 ± 2.74	278.00 ± 1.79

## Data Availability

Data available upon request.
